# Increasing salinity stress decreases the thermal tolerance of amphibian tadpoles in coastal areas of Taiwan

**DOI:** 10.1038/s41598-022-12837-7

**Published:** 2022-05-30

**Authors:** Ming-Feng Chuang, Yu-Jie Cheng, Desiree Andersen, Amaël Borzée, Chi-Shiun Wu, Yuan-Mou Chang, Yi-Ju Yang, Yikweon Jang, Yeong-Choy Kam

**Affiliations:** 1 Department of Life Sciences and Research Center for Global Change Biology, National Chung Hsing University, Taichung, 402202 Taiwan; 2 Department of Life Science, Tunghai University, Taichung, 407224 Taiwan; 3 Department of Life Sciences and Division of EcoScience, Ewha Womans University, Seoul, 03760 Republic of Korea; 4 Laboratory of Animal Behaviour and Conservation, College of Biology and the Environment, Nanjing Forestry University, Nanjing, 210037 China; 5 Department of Life Science, Chinese Culture University, Taipei, 111396 Taiwan; 6 Department of Ecology and Environmental Resources, National University of Tainan, Tainan, 700301 Taiwan; 7 Department of Natural Resources and Environmental Studies, National Dong Hwa University, Hualien, 974301 Taiwan; 8 Interdisciplinary Program of EcoCreative, Ewha Womans University, Seoul, 03760 Republic of Korea; 9 Department of Life Science, Tunghai University, Room LS320, No. 1727 Taiwan Boulevard, Taichung, 407224 Taiwan

**Keywords:** Climate-change ecology, Animal physiology, Herpetology

## Abstract

Global warming is the main cause for the rise of both global temperatures and sea-level, both major variables threatening biodiversity. Rising temperatures threaten to breach the thermal limits of organisms while rising sea-level threatens the osmotic balance of coastal animals through habitat salinization. However, variations in thermal tolerance under different salinity stresses have not yet been thoroughly studied. In this study, we assessed the critical thermal maxima (CTmax) of amphibian tadpoles in different salinity conditions. We collected tadpoles of *Duttaphrynus melanostictus*, *Fejervarya limnocharis* and *Microhyla fissipes* from coastal areas and housed them in freshwater, low, and high salinity treatments for 7 days of acclimation. The CTmax, survival rate, and development rate of tadpoles in high salinity treatments were significantly lower than that of the two other treatments. Our results indicate that physiological performances and heat tolerances of tadpoles are negatively affected by salinization. Maximum entropy models showed that CTmax and sea-level rise are predicted to negatively affect the distribution of the three focal species. The present results suggest that global warming can lead to negative dual-impacts on coastal animals because of reduced thermal tolerances at elevated salinity. The impacts of global warming on anurans in coastal areas and other habitats impacted by salinization may be more severe than predicted and it is likely to cause similar dual-impacts on other ectotherms.

## Introduction

Global warming leads to the thermal expansion of seawater and melting of polar ice caps, both major contributors to the rise of global mean sea level^1–4^. Between the late nineteenth century and the early twenty-first century, global mean sea level rose by 195 mm at an average velocity of 1.44 mm per year^5,6^. In addition, the global mean sea level is projected to rise by at least 1 m by the end of the twenty-first century^7,8^. Changes in sea level will result in negative ecological impacts such as habitat loss and salinization of coastal areas^9–12^.

Thermal tolerance varies greatly among species^13,14^, and the critical thermal maximum (CTmax) is a commonly applied index to assess the sensitivity of organisms to high temperature^15–25^. Earlier studies have evaluated the impact of warming temperature on species across latitudes using the more accurate warming tolerance (WT) index, defined as the differences between CTmax and the highest habitat temperature Tmax^13,14^. Results of earlier studies suggest that the impacts of rising temperature are more severe on species living in tropical areas when compared to temperate areas due to a narrower WT^13,14,26^. In addition, species with a lower CTmax are exposed to a greater risk in the presence of warming ambient temperature because of their smaller WT^13,26^.

Changes in environmental factors such as temperature have a greater impact on ectotherms, such as amphibians, which are present in a great variety of both aquatic and terrestrial habitats^27^. Amphibians are sensitive to environmental changes due to their permeable skin and their poor osmoregulatory ability during larval stages^27^, and especially so during the aquatic larvae life stage when they highly dependent on the availability of freshwater and humid environments^28,29^. In addition, rising water temperature is challenging the thermal tolerance of tadpoles and affecting their physiological function and ecological performance, including survival and development^14,30^.

Rising sea level can inundate land and cause saltwater to intrude into groundwater, resulting in habitat salinization, negatively affecting coastal amphibians. Earlier studies have reported that salinity exposure may decrease the survival and development of tadpoles^31–39^. However, acclimation to increasing salinity enhances salt tolerance of tadpoles by increasing the expression of branchial Na^+^, K^+^-ATPase (NKA)^34,36,40^. As a result, variation in salinity tolerance among amphibian species can directly influence the community composition, species abundance, and distribution^41^. Hence, global warming can affect both temperature and habitat salinity and both of these factors can affect organisms independently, but we know little about their joint effects.

Amphibians living and breeding in coastal areas may be more severely affected by global warming. Although amphibians generally avoid inhabiting and breeding in brackish habitats^42,43^, field surveys have revealed that some amphibian populations breed in this type of environment e.g. Refs.^44–48^. In addition, global warming leads to rising sea level, resulting in habitat salinization, potentially affecting the thermal tolerance of amphibians. Earlier studies have demonstrated that CTmax decreased in highly saline environments in several non-amphibian species^49–51^. However, it is still relatively unknown how atypical salinity affects thermal tolerance of animals and thus impacts their ecology, including the distribution of populations. In view of amphibians’ quality as early indicators of habitat condition changes^52^ and tadpoles’ specific sensitivity to water quality changes, amphibians are a perfect model to study thermal physiology in relation to salinity stress.

In this study, we used three anuran species: *Duttaphrynus melanostictus* (Bufonidae), *Fejervarya limnocharis* (Dicroglossidae), and *Microhyla fissipes* (Microhylidae). All of these species can be found in coastal areas, and *F. limnocharis* is known to breed in brackish habitats^53^. Based on the fact that salinity change has detrimental effects on thermal tolerance of vertebrates and invertebrates^49–51^, we predicted that the CTmax of tadpoles will decrease as salinity increases. In addition, earlier findings indicated that salinity may decrease survival and development rate of tadpoles^32–37^, and we consequently predict that the survival and development of tadpoles will be negatively affected by salinity.

## Materials and methods

### Animal collection and study sites

Tadpoles of *Duttaphrynus melanostictus*, *Fejervarya limnocharis* and *Microhyla fissipes* were used in this study. They are common species in lowlands of East Asia and breed from spring to summer, including July, the warmest month of the year. The monthly mean temperature, highest temperature, and lowest temperature recorded from nearest weather station were provided in the Supplemental Information (Supplementary Table S1; Supplementary Fig. S1). We collected tadpoles from coastal areas during summer, 2016. Tadpoles of *F. limnocharis* and *M. fissipes* were collected from the windbreak woodlands in Cheng-Xi village (23.041379° N, 120.080005° E) of Tainan County. The brackish tidal gullies where we collected tadpoles were covered with leaves and branches of *Casuarina equisetifolia*. The salinity fluctuation at that site depends on rainfall and evaporation^53^. When a typhoon comes, high tides bring seawater, and salinity dramatically increases from about one to eight ppt (*parts per thousand*). Tadpoles of *D. melanostictus* were collected from Lim-Chu (23.669155° N 120.175966° E) in the Yun-Lin County. Brackish water in this area was created by the seawater intrusion into groundwater caused by land subsidence^54^.

### Salinity acclimation

We collected 30–50 tadpoles at Gosner stages 26–30 for each species^55^ and brought them back to the laboratory. Tadpoles were subjected to three different treatments: freshwater, low salinity, and high salinity, with salinity based on their maximum salinity tolerance. For *D. melanostictus* and *F. limnocharis*, the low and high salinity treatments were 4 and 8 ppt, respectively^33^. For *M. fissipes*, the low and high salinity treatments were 3 and 5 ppt, respectively. The treatments for *M. fissipes* were based on a pilot study on salinity tolerance of tadpoles (see Supplementary Information). Tadpoles were randomly assigned to one of the three treatments (*n* = 10 to 15), with each tadpole reared separately in a 10.5 × 7.5 × 4.5 cm plastic container filled with 150 ml of the corresponding treatment solution. The freshwater solution used in this study was dechlorinated tap water, which was around 1 ppm. The salinity solution was a mixture of deionized water and Coralife sea salt (Energy Savers Unlimited, INC, Carson, CA, USA). Salinity was measured using an electronic salinity meter (Rixen, Model SM-10, Seoul, Republic of Korea) at room temperature. We did not use deionized water for control treatment due to the extreme low osmotic pressure, which may be harmful to tadpoles. Tadpoles were placed in an incubator and kept at 25 °C on a 12D:12L photoperiod.

### Effect of salinity on tadpole survival and development

Acclimation lasted for seven days, generally following the methods from Wu et al.^34^. We recorded the survival of tadpoles and renewed water every day to prevent the misalignment of salinity caused by evaporation and the deterioration of water quality caused by feeding. According to the specificity of foraging behaviour in each species, tadpoles of *F. limnocharis* and *D. melanostictus* were fed cooked spinach, while tadpoles of *M. fissipes* were fed powdery fish food daily. The developmental stages of tadpoles were measured before and after salinity acclimation. The developmental stages were identified based on Gosner criteria^55^. Each tadpole was isolated in a glass petri dish and the developmental stage was examined with a 10× hand lens and cold light.

### Critical thermal maximum measurement

The critical thermal maximum (CTmax) is defined as “the temperature at which an animal loses its essential motor function”^25^. CTmax of tadpoles was measured on the eighth day, right after acclimation. Each tadpole that survived during salinity acclimation was put into a beaker filled with 200 ml treatment water. The beaker was then put into a plastic box filled with water and then into a temperature-control water bath (BH-230D, Yihder Technology Co., ltd, Taiwan). Water was heated from ca. 25 °C at the rate of 0.25 °C/min^30^, while the water temperature was monitored by a probe thermal meter (nearest to 0.1 °C). We used a blunt probe to assess the activity of the tadpole every degree at the beginning of the experiment, and at increasing intervals every minute (ca. every 0.25° of increasing). We determined the CTmax as the point when a tadpole became disoriented in locomotion and lost its ability to escape from the stimuli. Once CTmax was reached, tadpoles were directly transferred into cool water for recovery.

### The expected distribution and coastal flooding area

Presence points from survey data of *D. melanostictus*, *F. limnocharis*, and *M. fissipes* were used in maximum entropy models (MaxEnt 3.4.4)^56^ for each species in Taiwan. The distribution data were collected every three months (four times in a year) by well-trained citizen scientists from 451 sites throughout Taiwan from 2006 to 2018, in which investigators did a 50 m transect visual encounter survey for each site, and resulting in a total of 28,601 observations for *D. melanostictus*, 26,625 observations for *F. limnocharis*, and 12,672 observations for *M. fissipes*. Nineteen bioclimatic variables (Supplementary Table S2) were used as predictor variables^57^ representing biologically meaningful climatic gradients. All variables were retained in models as is commonly used in MaxEnt modelling for other species^58–61^. A bias layer made from kernel density of all observation points was input into the model, and background points were set to 10,000. To reduce spatial bias, we selected the option in MaxEnt to remove duplicate presence points, effectively reducing all observations taken within the environmental layer cell size to a single occurrence when training the models. We used a regularization, or alpha, multiplier of three to smooth the responses of each species to bioclimatic variables. Each model was run for ten bootstrap replicates with a random test percentage of 20%. We used the averages of these replicates to represent the predicted suitability for each species across the Taiwan main island, with values above 0.5 representing predicted presence within a presence-absence dichotomy.

As a baseline of effect of salinity on CTmax, we first mapped the current maximum temperature across Taiwan using data from 29 weather stations from 2010 to 2018 (Central Weather Bureau of the Government of Taiwan), then classified by the mean CTmax calculations at different levels of salinity for each species and overlaid with current predicted distributions of each species. To create a uniform surface temperature layer across the study region, we used the ‘EBK regression prediction’ (Empirical Bayesian Kriging Regression Prediction) tool in ArcGIS Pro ver. 2.8.1 (ESRI, Redlands, CA, USA) with a digital elevation model (DEM) as our explanatory variable raster and maximum surface temperature at each georeferenced weather station as our dependent variable. In a one-week observation, the water temperature was within the daily variant range of air temperature, but lower the instant air temperature most of the time in study site Cheng-Xi village (see Supplementary Fig. S2).

To determine potential impact of climate change on the three tested species when the effect of salinity on CTmax was considered, we combined maps of 1 m sea level rise and predicted the maximum temperature for the year 2100 by increasing current temperature by 4.8 °C (Source: Airbus, USGS, NGA, NASA, CGIAR, GEBCO, NLS, OS, NMA, Geodatastyrelsen, GSA, GSI and the GIS User Community). Extrapolations to future climate change scenarios were created by adding projected increase in maximum temperature as constant values to our present-day temperature layer through the raster calculator. The predicted maximum temperature was also classified by CTmax (CTmax at no, low, and high salinity) and overlaid with current predicted distributions. All maps in this study were generated by using ArcMap 10.6 (ESRI, Redlands, CA, USA).

### Statistical analyses

The survival of tadpoles during acclimation was analyzed through a survival analysis (Cox proportional hazard model and Kaplan–Meier survival curve). Kruskal–Wallis tests with Dunn posterior tests were used for comparison between the developmental stage and CTmax of tadpoles acclimated in different treatments. Spearman’s rank correlation was used to test the effects of development stage on CTmax of tadpoles exposed to salinity treatments. Data analyses were conducted using R i386 3.1.0. Significance level was set as α = 0.05.

### Ethical statement

The animal use protocol in this study were carried out in accordance with relevant guidelines and regulations, and the experimental protocols have been reviewed and approved by the Institutional Animal Care and Use Committees of Tunghai University (IACUC permit No. 103-16), and all experiments were performed in accordance with relevant guidelines and regulations of ARRIVE Guidelines (https://arriveguidelines.org). No other permits were required because these field studies were not conducted in a protected area nor were our subjects an endangered or protected species. In total, there were 113 animals used as subjects in this study, 19 of them were sacrificed by the high salinity in the survival experiments, while there was no animal sacrificed doing the CTmax experiments. The animals were released at their site of capture right after finishing the experiments within 10–30 days of being captured.

## Results

### Effect of salinity on survival and development

**Figure 1:**
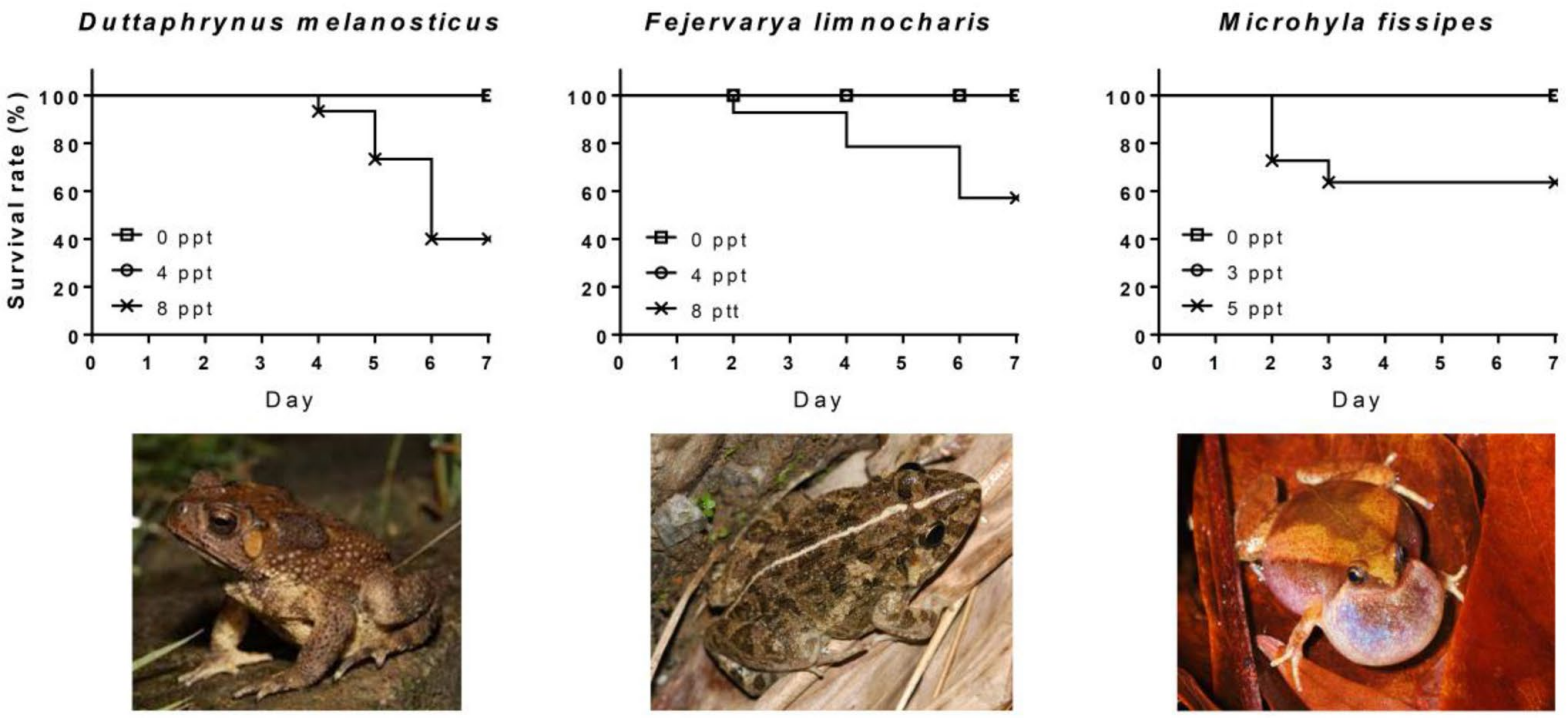
The survival rate of tadpoles under salty water. Survival of *Duttaphrynus melanostictus* (left), *Fejervarya limnocharis* (middle), and *Microhyla fissipes*(right) tadpoles in freshwater (open square), low salinity (open circle), and high salinity treatments (x-mark). *Duttaphrynus melanostictus*: *n* = 15, 14, and 15, respectively; *F. limnocharis*: *n* = 12, 12, and 14, respectively *M. fissipes*: *n* = 10, 10, and 11, respectively. (Photos were taken by M-F Chuang from Taichung City for *D. melanostictus*, and from Nantou County for *F. limnocharis* and *M. fissipes*.)

The survival analysis showed that the survival of tadpoles in all three species was significantly different among treatments of salinity (Cox model, likelihood ratio = 20.4, df = 2, *p* < 0.001 for *Duttaphrynus melanostictus*; likelihood ratio = 13.6, df = 2, *p* = 0.001 for *Fejervarya limnocharis*; and likelihood ratio = 9.12, df = 2, *p* = 0.011 for *Microhyla fissipes*, Fig. 1). No mortality occurred in freshwater and low salinity treatments in all three species. In the high salinity treatments, 57% of *F. limnocharis*, 60% of *D. melanostictus* and 64% *M. fissipes* tadpoles survived.

**Figure 2:**
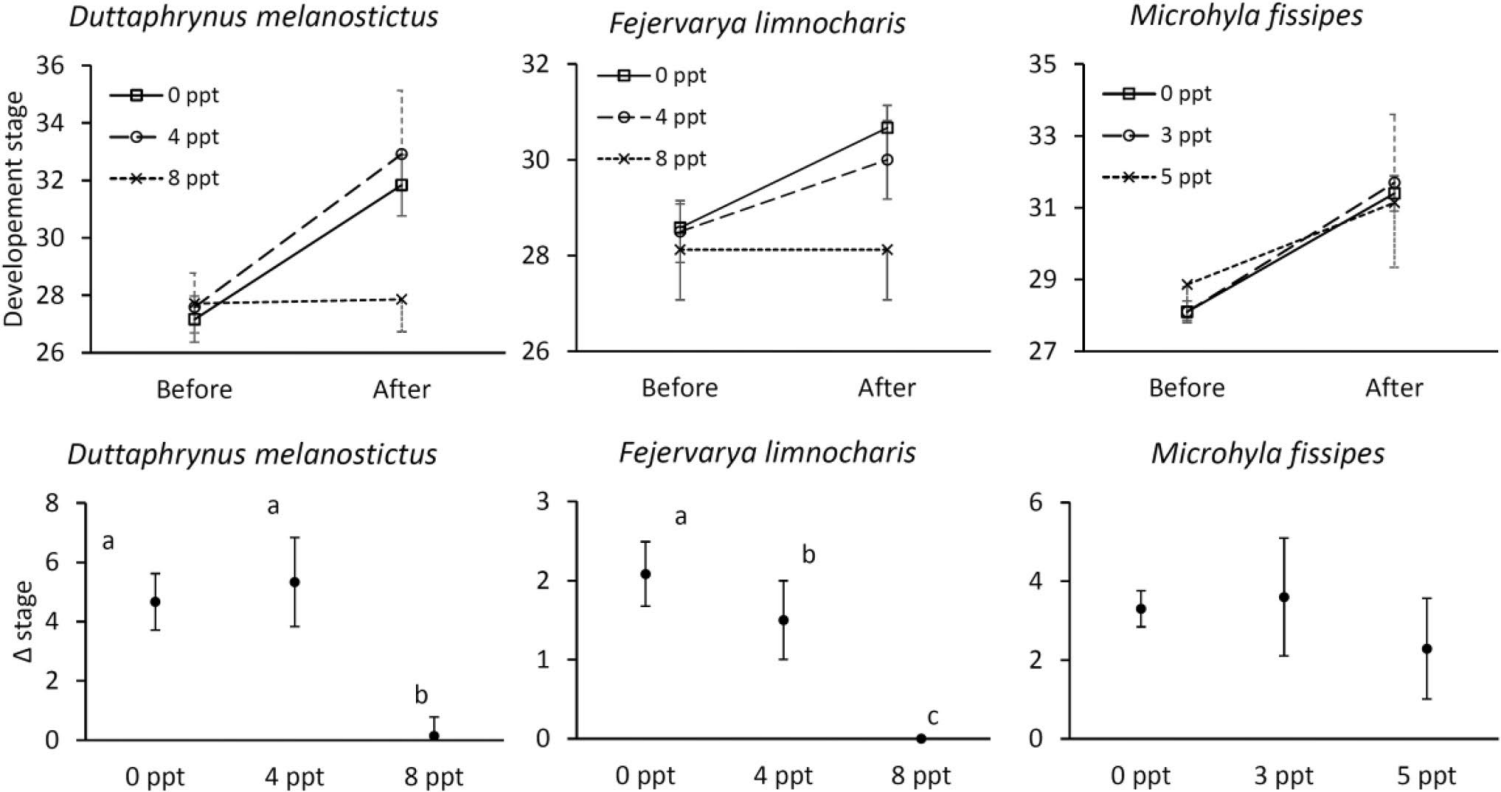
The development of tadpoles under salty water. The difference of developmental stage before and after salinity acclimation of *Duttaphrynus melanostictus* (left), *Fejervarya limnocharis* (middle), and *Microhyla fissipes* (right) tadpoles in freshwater (open square), low salinity (open circle), and high salinity treatments (x-mark). Values of Δ stage with different letters represent a statistical difference (Kruskal–Wallis tests with Dunn posterior tests, *p* < 0.05) among treatments. *Duttaphrynus melanostictus*: n = 12, 12, and 7, respectively; *Fejervarya limnocharis*: n = 12, 12, and 8, respectively; *Microhyla fissipes*: n = 10, 10, and 7, respectively. Data presented as means ± SD.

For all three species, the developmental stage of each species before acclimation was not significantly different between treatments (Kruskal–Wallis test, *F. limnocharis*: χ^2^ = 0.69, df = 2, *p* = 0.710; *D. melanostictus*: χ^2^ = 1.38, df = 2, *p* = 0.502; *M. fissipes*: χ^2^ = 4.04, df = 2, *p* = 0.133; Fig. 2). For *F. limnocharis,* there were significant differences in developmental stage between treatments after acclimation (Kruskal–Wallis test, χ^2^ = 19.31, df = 2, *p* < 0.001; Fig. 2 middle upper and bottom). Post hoc comparisons showed that development rate of tadpoles in freshwater was significantly faster than tadpoles in low (*p* = 0.001) and high salinity treatments (*p* < 0.001), and development rate of tadpoles in low salinity treatment was also significantly faster than in high salinity treatment (*p* = 0.008). Similarly, there were significant differences among treatments in development rate of *D. melanostictus* tadpoles (Kruskal–Wallis test, χ^2^ = 16.14, df = 2, *p* < 0.010; Fig. 2 left upper & bottom). Post hoc comparisons showed that there was no significant difference in development rates between freshwater and low salinity treatments (*p* = 0.285), and tadpoles in high salinity treatment developed significantly slower than those in freshwater (*p* < 0.001) and low salinity treatments (*p* < 0.001). The development rates of *M. fissipes* tadpoles were not significantly different among treatments (Kruskal–Wallis test, χ^2^ = 19.31, df = 2, *p* = 0.600; Fig. 2 right upper & bottom).

### Effect of salinity on CTmax

**Figure 3:**
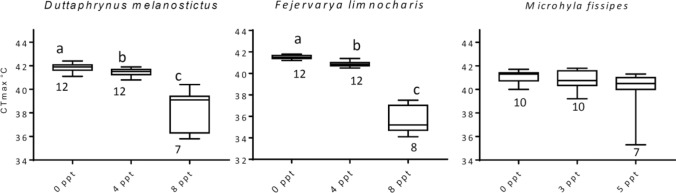
The critical thermal maximum (CTmax) of tadpoles under salty water. The CTmax of tadpoles of *Duttaphrynus melanostictus* (left), *Fejervarya limnocharis* (middle), and *Microhyla fissipes* (right) in freshwater, low and high salinity. The numbers below boxplot are sample sizes of each treatment. Data are presented as box and whisker plots. The horizontal line indicates the median value. Boxes indicate 25th and 75th percentiles; whiskers indicate minimum and maximum values. Values with different letters represent a statistical difference (Kruskal–Wallis tests with Dunn posterior tests, *p* < 0.05) among treatments.

Results showed that there were significant differences in CTmax among treatments in both *F. limnocharis* and *D. melanostictus* tadpoles (Kruskal–Wallis test, χ^2^ = 26.6, df = 2, *p* < 0.001; χ^2^ = 20.58, df = 2, *p* < 0.001, respectively). Post hoc comparison showed that the CTmax of *F. limnocharis* tadpoles in high salinity treatments was significantly lower than that of low salinity treatments (*p* = 0.008; Fig. 3 middle) and freshwater treatments (*p* < 0.001; Fig. 3 middle). Similarly, the CTmax of tadpoles in low salinity treatments was significantly lower than that of freshwater treatments (*p* = 0.001; Fig. 3 middle). There were similar results for *D. melanostictus* tadpoles, with CTmax for high salinity being significantly lower than that of low salinity (*p* = 0.004; Fig. 3 left) and freshwater treatments (*p* < 0.001; Fig. 3 left), while the CTmax in low salinity treatments was significantly lower than that in freshwater treatments (*p* = 0.015; Fig. 3 left). In contrast, we did not find a significant effect of salinity on CTmax of *M. fissipes* tadpoles (Kruskal–Wallis test, χ^2^ = 2.86, df = 2, *p* = 0.240; Fig. 3 right). In summary, all tested species showed a consistent pattern where CTmax of tadpoles decreased as salinity increased.

**Figure 4:**
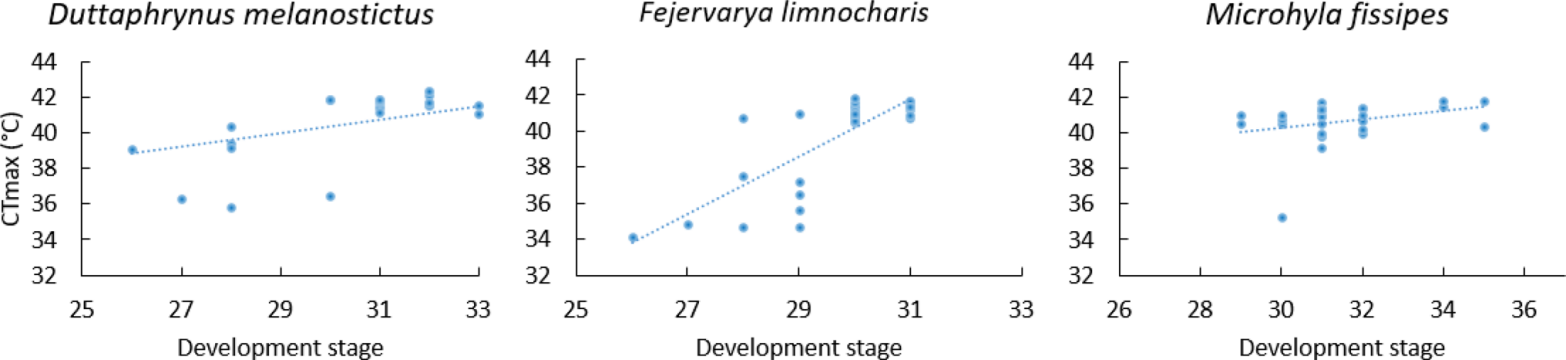
The critical thermal maximum (CTmax) of tadpoles under different development stage. The critical thermal maximum (CTmax) of tadpoles of *Duttaphrynus melanostictus* (Spearman’s r = 0.34, n = 31, p = 0.061; left), *Fejervarya limnocharis* (Spearman’s r = 0.75, n = 32, p < 0.001; middle), and *Microhyla fissipes* (Spearman’s r = 0.33, n = 27, p = 0.096; right) in different development stage.

In addition, we tested the effects of development stage on CTmax after exposed to salinity treatments. Results showed the CTmax of tadpoles was not in relation to development stage in *D. melanostictus* (Spearman’s r = 0.34, n = 31, p = 0.061; Fig. 4 left) and *M. fissipes* (Spearman’s r = 0.33, n = 27, p = 0.096; Fig. 4 right). In contrast, the CTmax of *F. limnocharis* tadpoles was in relation to the development stage after exposed to salinity treatments (Spearman’s r = 0.75, n = 32, p < 0.001; Fig. 4 middle).

### The expected distribution and coastal flooding area

**Figure 5:**
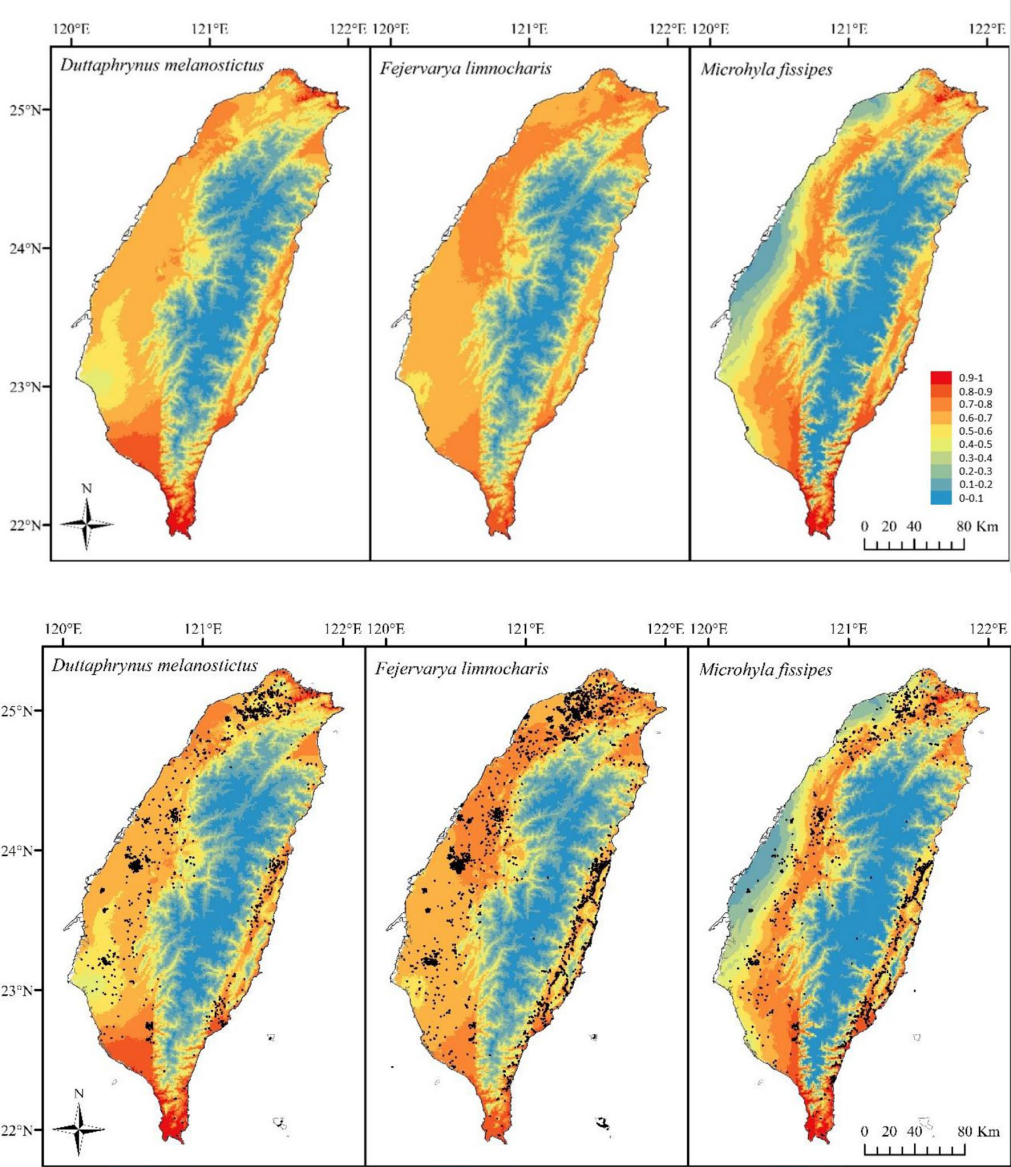
Suitability for three species (*Duttaphrynus melanostictus*, *Fejervarya limnocharis*, and *Microhyla fissipes*). Maps were modelled in MaxEnt using 19 bioclimatic variables^57^. Suitability ranges from low (blue) to high (red) with species primarily distributed in lowlands and coastal areas. Maps were generated by D. Andersen under ArcMap 10.6 (ESRI, Redlands, CA, USA).

MaxEnt models for the three species showed similar patterns of suitability across Taiwan (Fig. 5). Areas under the curve (AUC), or model fit, were 0.719, 0.734, and 0.771 for *D. melanostictus*, *F. limnocharis*, and *M. fissipes*, respectively. The area with a suitability over 0.5 covered 19,091 km^2^ for *D. melanostictus*, 20,811 km^2^ for *F. limnocharis*, and 15,340 km^2^ for *M. fissipes*. Suitable climate for *D. melanostictus* and *F. limnocharis* were found in low to middle elevations, while *M. fissipes* was distributed more broadly at intermediate elevations.

**Figure 6:**
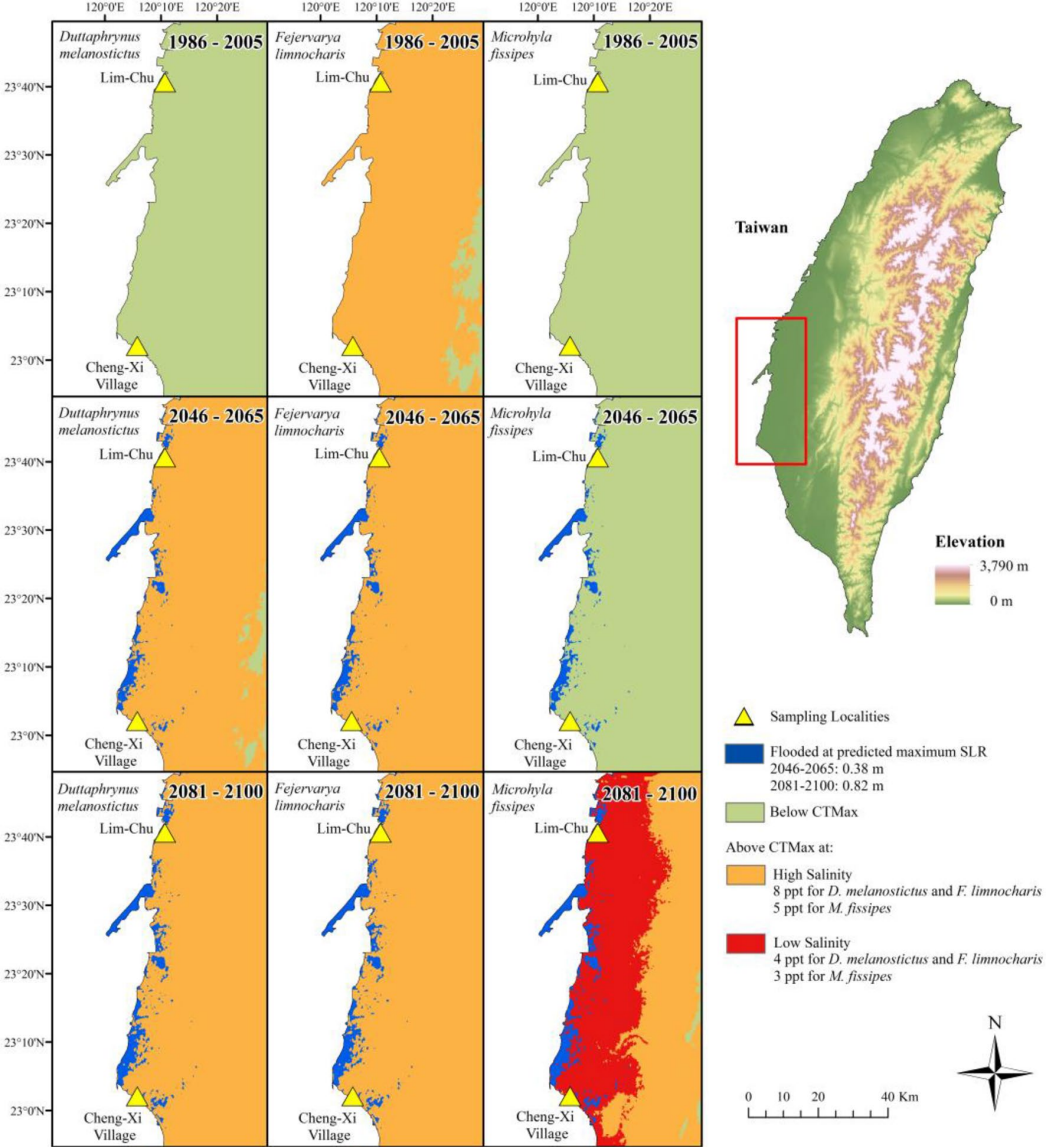
Current and future conditions for three species (*Duttaphrynus melanostictus*, *Fejervarya limnocharis*, and *Microhyla fissipes*). Conditions were compared by classifying maximum temperature into CTmax values at low and high salinity. While CTmax for high salinity is only reached for *F. limnocharis* at conditions for 1986–2005, climatic predictions for 2046–2065 indicate CTmax for high salinity will be reached for *D. melanostictus* and *F. limnocharis*. The climatic predictions for 2081–2100 indicate CTmax for high salinity will be reached for all species, and CTmax for low salinity will be reached for *M. fissipes*. Maps were generated by D. Andersen under ArcMap 10.6 (ESRI, Redlands, CA, USA).

Under the current climate, only *F. limnocharis* is predicted to occur in areas where Tmax is reaching CTmax for high salinity conditions (Fig. 6, top row). For year 2081–2100 predicted climate, however, high salinity CTmax temperatures will be reached for all species, and low salinity CTmax temperatures will be reached for *M. fissipes* (Fig. 6, bottom row). These areas also overlap with predicted flooding due to sea level rise (Fig. 6).

## Discussion

### Effect of salinity on survival and development

Survival and development rate of tadpoles were lower in high salinity treatments, highlighting that salinity has a negative effect on tadpole fitness. In this study, all three species exhibited a similar trend: lower survivals and delayed developments under increased salinity. Similar results were also reported in the same species of *Fejervarya limnocharis* tadpoles^33^, and also the tadpoles of *Litoria ewingii*^62^ and *Litoria aurea*^32^. Although development was not delayed obviously in *Microhyla fissipes* tadpoles, we suggested this may be due to the use of lower salinity as treatments for this species. Living in high salinity habitats not only leads to low survival, the delayed development of tadpoles may also increase the risks of predation or desiccation^63^ and result in a decreased population fitness.

### Effects of salinity on CTmax

The CTmax of tadpoles was lower when exposed to high salinity, suggesting that salinity reduces their ability to tolerate heat stress. The CTmax of *F. limnocharis* and *D. melanostictus* tadpoles acclimated in high salinity was significantly lower than that of tadpoles acclimated in freshwater, while there was no significant effect of salinity treatments on *M. fissipes* tadpoles. Our findings agree with results from earlier studies: CTmax of Antarctic collembolans (*Cryptopygus antarcticus*) acclimated in freshwater was 22.2 °C but dropped to 18.9 °C when acclimated in 200 ppt^51^. In addition, CTmax of yellow-fin sea breams (*Acanthopagrus latus*) maintained at 33 ppt was significantly lower than those in 0.3 and 15 ppt^49^, and CTmax of green sturgeons (*Acipenser medirostris*) acclimated in bay water (24 ppt) was significantly lower than those acclimated in freshwater 0–1 ppt^50^. However, the opposite pattern was found in three spine stickle back (*Gasterosteus aculeatus*), for which the CTmax of individuals acclimated in 20 ppt was significantly higher than that of individuals acclimated in 2 ppt^64^.

In contrast, the increase of salinity in hypotonic waters may increase CTmax. For instance, in the Andean toads (*Rhinella spinulosa*), froglets under 150 ppm (i.e. 0.15 ppt) salinity cannot tolerate more than 35 °C, whereas tadpoles in waters between 630 and 1800 ppm can tolerate temperatures higher than 36 °C^65^. As their results are opposite to ours, we assume there is an optimal salinity for animals, and CTmax will decrease in hypertonic or hypotonic solution^65^.

### Mechanisms of the decline of development and CTmax under salinity stress

The negative effects of high salinity on tadpoles is probably due to energy acquisition and allocation^66^ and the dehydration induced by salinity stress^51,67^. Several observations showed that tadpoles in high salinity water fed less^33,68,69^, suggesting that salinity negatively affected foraging behaviors, and thus resulted in a decrease in energy uptake to maintain regular development rate or CTmax. Salinity may depresses the thyroid hormone in tadpoles^70^, an essential hormone affecting their development^70,71^. Furthermore, previous studies showed that tadpoles consumed more glucose^70^ and had higher expression of branchial Na^+^/K^+^-ATPase (NKA) at high salinity^34,36^, suggesting that tadpoles spent more energy for osmoregulation and consequently reduced their development and ability to tolerate to temperature increase. A study also found that starving tadpoles had a significantly lower CTmax^22^, indicating that energy limitation had negative effect on the CTmax of tadpoles. In our study, we found the CTmax of at least one species was affected by development stage. However, the CTmax of tadpoles were usually steady throughout the larvae period^72^. Further experiments will be needed to figure out the relationships between these variables. For example, we noticed the food in high salinity treatment was usually left untouched. Hence, a quantitative experiment will help us to know the relationship between food intake and CTmax by the tadpoles acclimated in different salinity.

The decrease of CTmax may also relate to the dehydration of animals. Earlier studies showed that exposure to salinity could lead to dehydration at the cellular level^51,67^. *Fejervarya limnocharis* tadpoles are known to follow the same pattern, gradually dehydrating when transferred to a higher salinity treatment^34^. Earlier studies in other taxa showed that CTmax could be negatively affected by dehydration^73–75^. A proposed explanation is that cellular dehydration leads to changes in membrane fluidity, affecting heat tolerance^51^.

Finally, another explanation for why CTmax decreased for tadpoles acclimated in high salinity relates to the induction of heat shock proteins (HSPs). For instance, the increased CTmax at high salinity in *G. aculeatus* was associated with the induction of HSPs^64^. The heat shock response may decrease with time due to changes in HSP expression in response to nutritional status^76^. In this study, tadpoles acclimated for seven days may have starved, and consequently, protein re-allocation during starvation may have depressed the expression of HSPs, explaining why tadpoles displayed a lower CTmax under the high salinity treatments.

### The expected impact from simulation and the conservation issue

During the past two decades, the global amphibian decline has become an important conservation issue^77^, and numerous studies revealed that multiple causes contributed to the extirpation of amphibian population^78–80^. Our findings showed that the predicted impact of sea level rise in coastal regions may reduce suitable habitats due to inundation. In addition, underground saltwater intrusion, although not modeled in the present study, may further expand habitat loss of coastal freshwater habitats. Our findings that the CTmax of tadpoles declined in high salinity treatments has important implications for the effects of global warming on frog populations in coastal regions. Even though some amphibians are capable of surviving in brackish condition and adapted rapidly in several decades^81^, but species undergoing adaptive change may not do so at a pace to guarantee their persistence^82^. In regions where sea level rise is predicted to result in an increase in salinity levels of freshwater habitats, populations may become extirpated when the environmental salinity reaches the value of high salinity treatment, due to the reduction in survival and development as well as the reduction in thermal tolerance. Only the population of *M. fissipes* was predicted to be affected when environmental salinity reaches the value of low salinity treatment. However, the mean CTmax was fairly similar across the three treatments for *M. fissipes* suggesting that the species is not highly sensitive to salinization. Instead, the species is at greater risk due to its somewhat lower CTmax, regardless of salinity treatment. Also, the predictions based on air temperature may not accurately represent the real situation where tadpoles live, because tadpoles in deeper water, more shaded location, or by moderating temperature exposure behaviorally, may lower the impacts when the maximum air temperature reaches the organism's CTmax.

In conclusion, the results suggest that global warming has a double impact on the biology of tadpoles. During the larval period, rising temperature due to global warming gradually approaches the upper thermal limits of tadpoles, which threatens their survivorship^14^. The salinity stress not only retards the growth and development but also reduces the ability of tadpoles to tolerate high temperature. The distribution ranges of the three species in this study were also expected to shrink in the future. Global warming may negatively affect the survival and abundance of frogs more than we originally thought, particularly of populations in coastal areas. Our approach provides a novel perspective in explaining the global amphibian decline.

## Supplementary Information

Supplementary Tables.

Supplementary Figures.

## Data Availability

The datasets generated and/or analysed during the current study are available in the Supplemental Data.

## References

[1] Root TL (2003). Fingerprints of global warming on wild animals and plants. Nature.

[2] Meehl GA (2005). How much more global warming and sea level rise?. Science.

[3] Stocker, T. F. *et al.* (Cambridge University Press, 2013).

[4] Kopp RE (2014). Probabilistic 21st and 22nd century sea-level projections at a global network of tide-gauge sites. Earth's Future.

[5] Church, J. A. & White, N. J. A 20th century acceleration in global sea‐level rise. *Geophys. Res. Lett.* **33** (2006).

[6] Church JA, White NJ (2011). Sea-level rise from the late 19th to the early 21st century. Surv. Geophys..

[7] Vermeer M, Rahmstorf S (2009). Global sea level linked to global temperature. Proc. Natl. Acad. Sci..

[8] Horton BP, Rahmstorf S, Engelhart SE, Kemp AC (2014). Expert assessment of sea-level rise by AD 2100 and AD 2300. Quatern. Sci. Rev..

[9] Day JW, Pont D, Hensel PF, Ibañez C (1995). Impacts of sea-level rise on deltas in the Gulf of Mexico and the Mediterranean: The importance of pulsing events to sustainability. Estuaries.

[10] Feagin RA, Sherman DJ, Grant WE (2005). Coastal erosion, global sea-level rise, and the loss of sand dune plant habitats. Front. Ecol. Environ..

[11] Nicholls RJ (2011). Planning for the impacts of sea level rise. Oceanography.

[12] Hinkel J (2014). Coastal flood damage and adaptation costs under 21st century sea-level rise. Proc. Natl. Acad. Sci..

[13] Deutsch CA (2008). Impacts of climate warming on terrestrial ectotherms across latitude. Proc. Natl. Acad. Sci..

[14] Duarte H (2012). Can amphibians take the heat? Vulnerability to climate warming in subtropical and temperate larval amphibian communities. Glob. Change Biol..

[15] Licht P, Brown AG (1967). Behavioral thermoregulation and its role in the ecolgy of the red-bellied newt, *Taricha rivularis*. Ecology.

[16] Feder ME, Pough FH (1975). Temperature selection by the red-backed salamander, *Plethodon c. cinereus* (Green) (Caudata: Plethodontidae). Comp. Biochem. Physiol. Part A Physiol..

[17] Keen WH, Schroeder EE (1975). Temperature selection and tolerance in three species of *Ambystoma larvae*. Copeia.

[18] Hoppe, D. M. Thermal tolerance in tadpoles of the chorus frog *Pseudacris triseriata*. *Herpetologica*. 318–321 (1978).

[19] Cupp Jr, P. V. Thermal tolerance of five salientian amphibians during development and metamorphosis. *Herpetologica*. 234–244 (1980).

[20] Howard JH, Wallace RL, Stauffer JR (1983). Critical thermal maxima in populations of *Ambystoma macrodactylum* from different elevations. J. Herpetol..

[21] Floyd RB (1983). Ontogenetic change in the temperature tolerance of larval *Bufo marinus* (Anura: Bufonidae). Comp. Biochem. Physiol. A Physiol..

[22] Floyd RB (1985). Effects of photoperiod and starvation on the temperature tolerance of larvae of the giant toad, *Bufo marinus*. Copeia.

[23] Manis ML, Claussen DL (1986). Environmental and genetic influences on the thermal physiology of *Rana sylvatica*. J. Therm. Biol.

[24] Layne J, Claussen D, Manis M (1987). Effects of acclimation temperature, season, and time of day on the critical thermal maxima and minima of the crayfish *Orconectes rusticus*. J. Therm. Biol.

[25] Lutterschmidt WI, Hutchison VH (1997). The critical thermal maximum: History and critique. Can. J. Zool..

[26] Simon MN, Ribeiro PL, Navas CA (2015). Upper thermal tolerance plasticity in tropical amphibian species from contrasting habitats: Implications for warming impact prediction. J. Therm. Biol.

[27] Boutilier R, Donohoe P, Tattersall G, West T (1997). Hypometabolic homeostasis in overwintering aquatic amphibians. J. Exp. Biol..

[28] Shoemaker V, Nagy KA (1977). Osmoregulation in amphibians and reptiles. Annu. Rev. Physiol..

[29] Viertel B (1999). Salt tolerance of *Rana temporaria*: Spawning site selection and survival during embryonic development (Amphibia, Anura). Amphibia-Reptilia.

[30] Wu C-S, Kam Y-C (2005). Thermal tolerance and thermoregulation by Taiwanese rhacophorid tadpoles (*Buergeria japonica*) living in geothermal hot springs and streams. Herpetologica.

[31] Gomez-Mestre I, Tejedo M (2003). Local adaptation of an anuran amphibian to osmotically stressful environments. Evolution.

[32] Christy MT, Dickman CR (2002). Effects of salinity on tadpoles of the green and golden bell frog (*Litoria aurea*). Amphibia-Reptilia.

[33] Wu C-S, Kam Y-C (2009). Effects of salinity on the survival, growth, development, and metamorphosis of *Fejervarya limnocharis* tadpoles living in brackish water. Zool. Sci..

[34] Wu CS, Yang WK, Lee TH, Gomez-Mestre I, Kam YC (2014). Salinity acclimation enhances salinity tolerance in tadpoles living in brackish water through increased Na+, K+-ATPase expression. J. Exp. Zool. A Ecol. Genet. Physiol..

[35] Alexander LG, Lailvaux SP, Pechmann JH, DeVries PJ (2012). Effects of salinity on early life stages of the Gulf Coast toad, *Incilius nebulifer* (Anura: Bufonidae). Copeia.

[36] Bernabò I, Bonacci A, Coscarelli F, Tripepi M, Brunelli E (2013). Effects of salinity stress on *Bufo balearicus* and *Bufo bufo* tadpoles: Tolerance, morphological gill alterations and Na+/K+-ATPase localization. Aquat. Toxicol..

[37] Kearney BD, Pell RJ, Byrne PG, Reina RD (2014). Anuran larval developmental plasticity and survival in response to variable salinity of ecologically relevant timing and magnitude. J. Exp. Zool. A Ecol. Genet. Physiol..

[38] Hsu WT, Wu CS, Hatch K, Chang YM, Kam YC (2018). Full compensation of growth in salt-tolerant tadpoles after release from salinity stress. J. Zool..

[39] Hsu W-T (2012). Salinity acclimation affects survival and metamorphosis of crab-eating frog tadpoles. Herpetologica.

[40] Lai J-C, Kam Y-C, Lin H-C, Wu C-S (2019). Enhanced salt tolerance of euryhaline tadpoles depends on increased Na+, K+-ATPase expression after salinity acclimation. Comp. Biochem. Physiol. A: Mol. Integr. Physiol..

[41] Brown ME, Walls SC (2013). Variation in salinity tolerance among larval anurans: Implications for community composition and the spread of an invasive, non-native species. Copeia.

[42] Balinsky JB (1981). Adaptation of nitrogen metabolism to hyperosmotic environment in Amphibia. J. Exp. Zool. A Ecol. Genet. Physiol..

[43] Duellman W, Trueb L (1994). Biology of Amphibians.

[44] Alcala AC (1962). Breeding behavior and early development of frogs of Negros, Philippine Islands. Copeia.

[45] Gordon MS, Tucker VA (1965). Osmotic regulation in the tadpoles of the crab-eating frog (*Rana cancrivora*). J. Exp. Biol..

[46] Dunson WA (1977). Tolerance to high temperature and salinity by tadpoles of the Philippine frog, *Rana cancrivora*. Copeia.

[47] Uchiyama M, Murakami T, Wakasugi C, Yoshizawa H (1990). Structure of the kidney in the crab-eating frog, *Rana cancrivora*. J. Morphol..

[48] Heo K, Kim YI, Bae Y, Jang Y, Borzée A (2019). First report of *Dryophytes japonicus* tadpoles in saline environment. Russ. J. Herpetol..

[49] Jian CY, Cheng SY, Chen JC (2003). Temperature and salinity tolerances of yellowfin sea bream, *Acanthopagrus latus*, at different salinity and temperature levels. Aquac. Res..

[50] Sardella BA, Sanmarti E, Kültz D (2008). The acute temperature tolerance of green sturgeon (*Acipenser medirostris*) and the effect of environmental salinity. J. Exp. Zool. A Ecol. Genet. Physiol..

[51] Everatt MJ, Worland MR, Convey P, Bale JS, Hayward SA (2013). The impact of salinity exposure on survival and temperature tolerance of the Antarctic collembolan *Cryptopygus antarcticus*. Physiol. Entomol..

[52] Kerby JL, Richards-Hrdlicka KL, Storfer A, Skelly DK (2010). An examination of amphibian sensitivity to environmental contaminants: are amphibians poor canaries?. Ecol. Lett..

[53] Chang YM, Wu CS, Huang YS, Sung SM, Hwang W (2016). Occurrence and reproduction of anurans in brackish water in a coastal forest in Taiwan. Herpetol. Notes.

[54] Peng TR, Hsieh YH, Liu TS (2005). Hydro chemical characteristics and salinization of groundwater in Yunlin area. J. Chin. Soil Water Conserv..

[55] Gosner KL (1960). A simplified table for staging anuran embryos and larvae with notes on identification. Herpetologica.

[56] Phillips SJ, Anderson RP, Dudík M, Schapire RE, Blair ME (2017). Opening the black box: An open-source release of Maxent. Ecography.

[57] Hijmans RJ, Cameron SE, Parra JL, Jones PG, Jarvis A (2005). Very high resolution interpolated climate surfaces for global land areas. Int. J. Climatol..

[58] Groff LA, Marks SB, Hayes MP (2014). Using ecological niche models to direct rare amphibian surveys: A case study using the Oregon Spotted Frog (*Rana pretiosa*). Herpetol. Conserv. Biol..

[59] Kumar P (2012). Assessment of impact of climate change on *Rhododendrons* in Sikkim Himalayas using Maxent modelling: limitations and challenges. Biodivers. Conserv..

[60] Pineda E, Lobo JM (2009). Assessing the accuracy of species distribution models to predict amphibian species richness patterns. J. Anim. Ecol..

[61] Yuan H-S, Wei Y-L, Wang X-G (2015). Maxent modeling for predicting the potential distribution of Sanghuang, an important group of medicinal fungi in China. Fungal Ecol..

[62] Chinathamby K, Reina RD, Bailey PC, Lees BK (2006). Effects of salinity on the survival, growth and development of tadpoles of the brown tree frog, *Litoria ewingii*. Aust. J. Zool..

[63] Metcalfe NB, Monaghan P (2001). Compensation for a bad start: Grow now, pay later?. Trends Ecol. Evol..

[64] Metzger DC, Healy TM, Schulte PM (2016). Conserved effects of salinity acclimation on thermal tolerance and hsp70 expression in divergent populations of threespine stickleback (*Gasterosteus aculeatus*). J. Comp. Physiol. B..

[65] Sanabria E (2018). Effect of salinity on locomotor performance and thermal extremes of metamorphic Andean Toads (*Rhinella spinulosa*) from Monte Desert, Argentina. J. Therm. Biol..

[66] Sokolova IM (2013). Energy-limited tolerance to stress as a conceptual framework to integrate the effects of multiple stressors. Integr. Comp. Biol..

[67] Kikawada T (2006). Dehydration-induced expression of LEA proteins in an anhydrobiotic chironomid. Biochem. Biophys. Res. Commun..

[68] Sanzo D, Hecnar SJ (2006). Effects of road de-icing salt (NaCl) on larval wood frogs (*Rana sylvatica*). Environ. Pollut..

[69] Wood L, Welch AM (2015). Assessment of interactive effects of elevated salinity and three pesticides on life history and behavior of southern toad (*Anaxyrus terrestris*) tadpoles. Environ. Toxicol. Chem..

[70] Gomez-Mestre I, Tejedo M, Ramayo E, Estepa J (2004). Developmental alterations and osmoregulatory physiology of a larval anuran under osmotic stress. Physiol. Biochem. Zool..

[71] Dent JN (1988). Hormonal interaction in amphibian metamorphosis 1 2. Am. Zool..

[72] Bodensteiner BL (2021). Thermal adaptation revisited: How conserved are thermal traits of reptiles and amphibians?. J. Exp. Zool. Part A Ecol. Integr. Physiol..

[73] Rezende EL, Tejedo M, Santos M (2011). Estimating the adaptive potential of critical thermal limits: Methodological problems and evolutionary implications. Funct. Ecol..

[74] Mitchell JD, Hewitt P, Van Der Linde TDK (1993). Critical thermal limits and temperature tolerance in the harvester termite *Hodotermes mossambicus* (Hagen). J. Insect Physiol..

[75] Plummer MV, Williams BK, Skiver MM, Carlyle JC (2003). Effects of dehydration on the critical thermal maximum of the desert box turtle (*Terrapene ornata luteola*). J. Herpetol..

[76] Lee S (2016). Effects of feed restriction on the upper temperature tolerance and heat shock response in juvenile green and white sturgeon. Comp. Biochem. Physiol. A Mol. Integr. Physiol..

[77] Blaustein AR, Wake DB (1990). Declining amphibian populations: A global phenomenon?. Trends Ecol. Evol..

[78] Kiesecker JM, Blaustein AR, Belden LK (2001). Complex causes of amphibian population declines. Nature.

[79] Rohr JR, Raffel TR (2010). Linking global climate and temperature variability to widespread amphibian declines putatively caused by disease. Proc. Natl. Acad. Sci..

[80] Pounds JA (2006). Widespread amphibian extinctions from epidemic disease driven by global warming. Nature.

[81] Skelly D, Freidenburg L (2000). Effects of beaver on the thermal biology of an amphibian. Ecol. Lett..

[82] Radchuk V (2019). Adaptive responses of animals to climate change are most likely insufficient. Nat. Commun..

