# Supplementary material for: Increasing salinity stress decreases the thermal tolerance of amphibian tadpoles in coastal areas of Taiwan
Source: Sci Rep. 2022 May 30;12:9014. doi: 10.1038/s41598-022-12837-7 (PMC9151724; doi:10.1038/s41598-022-12837-7)
Supplement: Supplementary file 2 — Supplementary Figures. [file 41598_2022_12837_MOESM2_ESM.docx]

# Increasing salinity stress decreases the thermal tolerance of amphibian tadpoles in coastal areas of Taiwan

Ming-Feng Chuang, Yu-Jie Cheng, Desiree Andersen, Amaël Borzée, Chi-Shiun Wu, Yuan-Mou Chang, Yi-Ju Yang, Yikweon Jang, Yeong-Choy Kam

**Supplemental information 1**

*A test on the salinity tolerance of Microhyla fissipes*

The aim of this pretest was to measure the salinity tolerance of tadpoles. A total of 50 tadpoles at Gosner stage 26-30 (Gosner 1960) were collected from the windbreak woodlands in Cheng-Xi village of Tainan County and brought back to the laboratory in the summer of 2015. Tadpoles were put into 10 different salinity treatments (1, 2, 3, 4, 5, 6, 7, 8, 9, 10 ppt) for 48 hours (*n*=5). Each tadpole was kept in a 10.5 × 7.5 × 4.5 cm plastic container filled with 150 ml corresponding solution. Tadpole survivorship was checked every hour for the first 12 hours and then at the 24^th^ and 48^th^ hour.

No mortality was recorded at 1, 2, 3, and 4 ppt at all check points. In contrary, two tadpoles died in 5 ppt and three tadpoles died in 6 ppt before 48 hours. Moreover, tadpoles died within a day when salinity exceeded 7 ppt. Based on these results, we assumed that 6 ppt was the upper salinity limit for *M. fissipes* tadpoles for the acclimation experiment. Thus, we set the high salinity treatment at 5 ppt as the sub-lethal concentration and the low salinity at 3 ppt.

**Supplemental information 2**

*The history monthly temperature*

**Table S1**. The history monthly temperature (°C) in Tainan Weather Station (recorded from 1991-2020, Taiwan Central Weather Bureau)

| The monthly temperature (°C) in Tainan Weather Station (recored from 1991-2020, Taiwan Central Weather Bureau) | | | | | | | | | | | | |
| --- | --- | --- | --- | --- | --- | --- | --- | --- | --- | --- | --- | --- |
| Month | Jan | Feb | Mar | Apr | May | Jun | Jul | Aug | Sep | Oct | Nov | Dec |
| Tmax | 22.9 | 24 | 26.5 | 29.3 | 31.4 | 32.4 | 33.1 | 32.6 | 32.5 | 30.8 | 28.1 | 24.4 |
| Tmean | 17.8 | 18.9 | 21.6 | 24.9 | 27.5 | 28.9 | 29.4 | 28.9 | 28.6 | 26.3 | 23.4 | 19.6 |
| Tmin | 14.5 | 15.5 | 18 | 21.7 | 24.7 | 26.3 | 26.7 | 26.3 | 26 | 23.3 | 20.3 | 16.3 |

**Figure S1**. The history monthly temperature (°C) in Tainan Weather Station (recorded from 1991-2020, Taiwan Central Weather Bureau).

**Supplemental information 3**

*The description of bioclimatic variables*

**Table S2**. The bioclimatic variables. The bioclimatic variables represent biologically meaningful variables that are often used in species distribution modeling. There are 19 variables and are described as below:

| Bioclimatic variables | Description |
| --- | --- |
| BIO1 | Annual Mean Temperature |
| BIO2 | Mean Diurnal Range (Mean of monthly (max temp - min temp)) |
| BIO3 | Isothermality (BIO2/BIO7) (* 100) |
| BIO4 | Temperature Seasonality (standard deviation *100) |
| BIO5 | Max Temperature of Warmest Month |
| BIO6 | Min Temperature of Coldest Month |
| BIO7 | Temperature Annual Range (BIO5-BIO6) |
| BIO8 | Mean Temperature of Wettest Quarter |
| BIO9 | Mean Temperature of Driest Quarter |
| BIO10 | Mean Temperature of Warmest Quarter |
| BIO11 | Mean Temperature of Coldest Quarter |
| BIO12 | Annual Precipitation |
| BIO13 | Precipitation of Wettest Month |
| BIO14 | Precipitation of Driest Month |
| BIO15 | Precipitation Seasonality (Coefficient of Variation) |
| BIO16 | Precipitation of Wettest Quarter |
| BIO17 | Precipitation of Driest Quarter |
| BIO18 | Precipitation of Warmest Quarter |
| BIO19 | Precipitation of Coldest Quarter |

**Supplemental information 4**

*The water temperature & air temperature in study site*

**Figure S2**. The water temperature and air temperature in Cheng-Xi village (23.041379°N, 120.080005°E), recorded from Nov 2^nd^-8^th^, 2021 by two water proof temperature data loggers (Hobo Pendant Temperature Data Logger UA-001, Onset Computer Corporation, MA 02532, USA). One logger set on a tree with c.a. 1.5 m above the ground, the other set in the water with c.a. 30 cm depth. Sites of both data loggers did not receive sunlight directly.
